# Cardiac Critical Care: From Past Achievements to Future Advances

**DOI:** 10.14797/mdcvj.1275

**Published:** 2023-08-01

**Authors:** Faisal N. Masud

**Affiliations:** 1 Houston Methodist DeBakey Heart & Vascular Center, Houston Methodist Hospital, Houston, Texas, US

**Keywords:** cardiac critical care, ICU, vICU

In its second century as a medical specialty, critical care was thrust into the spotlight when patients with COVID-19 began appearing in emergency rooms and filling intensive care units in March 2020. The ensuing months, which became years, revealed the impressive strengths and humbling vulnerabilities of managing an unprecedented global crisis. While years of planning and preparation helped make the challenges manageable, a major revelation for many was the extent of interconnection across body systems and between various organs, especially the heart. This clarity in vision has spurred great advances in critical care, including new research and therapies for patients, particularly in the cardiac intensive care units (ICU) and cardiothoracic ICU.

In this issue of the *Methodist DeBakey Cardiovascular Journal*, we explore the topic of “Cardiac Critical Care” and its evolution over the past 3.5 years. We begin our exploration with “Integrating a Virtual ICU with Cardiac and Cardiovascular ICUs.” Drs. Atiya Dhala, Mario V. Fusaro, and coauthors share their journey of establishing Houston Methodist’s tele-critical care program, also known as our Virtual ICU or vICU. Several years in the making, our vICU’s fortuitous launch early in 2020 occurred just in time to support the challenges brought by COVID-19. Here, we offer a prescriptive roadmap for planning, launching, and integrating vICU services within cardiac and cardiovascular ICUs—among the first integrations in the United States (US). Among the many benefits, our data reflect fewer night-call requirements for in-person intensivists, increased work satisfaction, and reduced rates of “Code Blue” events.

Looking to the next generation of emerging technologies, coauthors Peter Osztrogonacz, Alan Lumsden, and Ponraj Chinnadurai share a first glance of implementing artificial intelligence and telemedicine to reshape patient care. In “Emerging Applications for Remote Video Surveillance and Artificial Intelligence on the Management of the CV Patient,” the authors describe initiatives that are already underway, including fall detection and prevention, automation of instrument counts in the operating room, and efficiency optimization in the cardiovascular suite.

Another opportunity for rethinking the future comes with new approaches to managing blood pressure. In “Blood Pressure Goals in Critically Ill Patients,” Drs. Khanna, Karuna Puttur Rajkumar, and colleagues call into question conventional blood pressure targets for ICUs. They propose that perfusion pressure may be a more important target than mean pressure, and they advocate for individualized blood pressure targets tailored to specific cardiac pathophysiology and patient characteristics.

In “Vasoplegia: A Review,” Drs. Ratnani, Hafsa Nazir Jatoi, and colleagues focus on this form of vasodilatory shock, which may occur as a complication of cardiac surgery in up to 25% of patients. Identification of multiple risk factors can support prompt management of vasoplegia to prevent development of shock. While describing the different vasopressors and other agents used to manage vasoplegia, the authors also emphasize the need for further research that will advance treatment regimens.

With more than 400,000 cardiac implantable electronic devices implanted annually in the US, infection is a serious complication of these procedures, associated with high morbidity and mortality. “Clinical Approach to Evaluation of Underlying Cardiac Device Infection in Patients Hospitalized with Bacteremia” reviews current data in the light of newly updated 2023 Duke-ISCVID criteria for infective endocarditis. Coauthors Deirdre Axell-House, Sarwat Khalil, and Muhammad Rizwan Sohail also include an algorithm offering an overview for diagnostic evaluation of CIED infection in patients presenting with bacteremia.

“Acute Respiratory Distress Syndrome in Patients with Cardiovascular Disease” by Drs. Asma Zainab, Megan Gooch, and Divina Tuazon offers an insightful review of the cause-and-effect relationship between cardiovascular disease and acute respiratory distress syndrome (ARDS), as revealed in the literature over the past 25 years. As the disease burden of both conditions has both a national and global impact on health care, the authors emphasize the importance of identifying underlying causes and phenotypes of ARDS while providing individualized care within algorithm-based ARDS and cardiac care programs.

Exploring another form of shock, Drs. Bassel Akbik, Philip Chou, and Janardhana Gorthi write about “Extracorporeal Membrane Oxygenation Use in Post-Cardiotomy Cardiogenic Shock.” While post-cardiotomy extracorporeal membrane oxygenation (ECMO) is increasingly used as the first-line mechanical circulatory support in patients who are refractory to conventional treatment, limited evidence is available regarding its safety, efficacy, and optimal timing for initiation and weaning. This summary shares current evidence on ECMO use in post-cardiotomy cardiogenic shock, and discusses its potential benefits, management, complications, and outcomes.

Clinicians caring for critically ill patients with cardiac disease must understand the complex syndrome of ICU delirium and recognize its impact in predicting long-term outcomes for ICU patients. While offering a comprehensive review in “ICU Delirium in Cardiac Patients,” Drs. Hina Faisal, Souha Farhat, and coauthors highlight the presentation, course, risk factors, pathophysiology, and management of the condition. They also examine controversies and future considerations of innovative therapies, including pharmacological as well as other management interventions.

With a final look to future advancements, Dr. Raul Sanchez Leon and coauthors Anjana Rajaraman and Mitzi Kubwimana bring knowledge of preoperative nutritional status and its effect on postsurgical outcomes. In “Optimizing Nutritional Status of Patients Prior to Major Surgical Intervention,” they share evidence-based perioperative nutrition guidelines, including enhanced recovery protocols and nutritional support recommendations, as initiatives for improving surgery-focused goals in patient care.

While we have seen tremendous achievements and progress in just a few short years, we continually seek future advancement. Among our long-standing challenges, which have only intensified since the COVID-19 pandemic, is the shortage of critical care intensivists and nurses. As we look to the future, we explore technological solutions for improving our work environment while also expanding and advancing intensive care practices for our patients.

For all of their amazing insights, I thank our authors and reviewers, who have shared their expertise and provided up-to-date views on issues that continue to bring greater advancements to our field and for our patients.

## Guest Editor Biography

The editors of the *Methodist DeBakey Cardiovascular Journal* express our appreciation to Dr. Faisal N. Masud for his guidance, expertise, and dedication in curating this issue on cardiac critical care.

## Faisal N. Masud, MD, FCCM, FCCP

**Figure:**
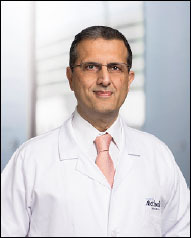


Dr. Faisal Masud has served as Board Member, Houston Methodist Board of Directors, and Medical Director of Center for Critical Care at Houston Methodist Hospital System, overseeing eight hospital ICUs and vICUs. He also serves as Vice Chair for Quality and Patient Safety, and is the Associate Quality Officer. He is a professor of Clinical Anesthesiology, Weill Cornell Medical College, professor of Anesthesiology at Houston Methodist Institute of Academic Medicine, professor in the Department of Acute & Continuing Care at UT-Houston, and adjunct clinical professor at Texas A&M School of Medicine. He received his training at Duke University Medical Center and Cleveland Clinic Foundation.

Among his many awards are the Presidential Gold Medal, Golden Apple Award for Excellence in Teaching (four times), the Dean H. Morrow Resident Mentor Award (2001), and the prestigious Fulbright & Jaworski Faculty Excellence Award in Educational Leadership.

Dr. Masud’s leadership has led to great improvements in patient care and clinical outcomes, safety & quality, sepsis control, central line infections, ventilator-associated pneumonia, surgical site infections, and blood transfusions, resulting in hundreds of lives saved and millions of dollars in cost savings. He also focuses on improving health care in underdeveloped health systems in less developed countries and has done volunteer work to improve health care in vulnerable areas for many years.

